# Utilising the Orthoptic Skill Set to Improve Access to Eye Care for Adults with Severe/Profound Learning Disabilities–A Service Evaluation

**DOI:** 10.22599/bioj.162

**Published:** 2021-03-10

**Authors:** Kathy Diplock, Jignasa Mehta

**Affiliations:** 1 Torbay and South Devon NHS Foundation Trust, GB; 2 University of Liverpool, GB

**Keywords:** orthoptist, adults with learning disabilities, visual impairment, eye care pathway, multidisciplinary team

## Abstract

**Intro:**

There is a wealth of research evidencing the high incidence of visual impairment (VI) and poor uptake of eye care services by adults with learning disabilities (LD). Despite this, very few authorities within England currently provide the additional support required by those with severe/profound LD (S/PLD).

**Method:**

By means of an initial funded pilot study, an unmet need was evidenced locally and a small service established to improve access to eye care for adults with S/PLD. Operational since 2007, this service has provided evidence to support the campaign for a nationally funded eye care pathway.

This service evaluation outlines the initial service set up, aims and objectives, and provides an analysis of the current service by means of a detailed breakdown of service-user outcomes during a sample 5-year period.

**Results:**

Orthoptic home visits (OHV) revealed high levels of strabismus (54.4%), refractive error (43.3%), cataracts (23.3%), and many other ophthalmic conditions (29%). Over a quarter of the adults with LD (26.6%) were certified as VI and 61% of people were provided with tailored strategies, the majority of which were for visual processing difficulties.

**Conclusion:**

The prospect of a nationally funded eye care pathway for adults with LD in England is now a real possibility. This service model has identified a clearly defined unmet need and illustrates the unique skill set orthoptists can offer to address this health inequality. Requiring minimal financial outlay and flexible enough to be integrated into any future national eye care framework, this service has ensured that access to eye care is truly equitable for all people with LD.

## Introduction

A significant body of research has been accrued over the past 50 years evidencing the much higher incidence of visual impairment (VI) and ocular defects in people with learning disabilities (LD). Studies pre-2001 are summarised in the review paper by Warburg (2001a). These findings led to the consensus statement of the International Association for the Scientific Study of Intellectual Disabilities (IASSID) in 1998 detailing guidelines for the active detection and diagnosis of VI for people with LD (Evenhuis and Nagtzaam, 1998).

In the general adult population of Europe, the rate of VI is reported as 0.6%, for blindness 0.1–3.9% (Buch et al., 2004; Kocur and Resnikoff, 2002), with the highest rates being amongst older people where age-related macular degeneration and cataract were the most common cause of VI. Van Splunder et al. (2004) noted that for people with LD, age, the severity of the LD, and the presence of Down syndrome (DS) all resulted in significant increases in the rates of VI and blindness, ranging from 2% for young people with mild LD and no DS (van Splunder et al., 2006) to 92%–100% for older people with severe/profound LD (S/PLD) (van den Broek et al., 2006; McCulloch et al., 1996). As people with S/PLD comprise a larger than expected proportion of the overall LD population, van Splunder et al. (2006) suggested that these people should be considered VI until proven otherwise. Studies suggest the most common causes of VI for people with LD were uncorrected/uncorrectable refractive error (25–61%), cataract (7–42%), keratoconus (2–3%), and optic atrophy (2–11%) (Krinsky-McHale et al., 2012; van Splunder et al., 2003; van Splunder et al., 2004; van Splunder et al., 2006; Evenhuis et al., 2001; Warburg, 2001b; Woodhouse, Griffiths & Gedling, 2000; McCulloch et al., 1996). Cerebral visual impairment (CVI) formed the most common untreatable disorder (12%–47%) (van Splunder et al., 2004; Evenhuis et al., 2001).

In the UK, vision monitoring has been provided for children in special schools for many years but only in some areas and despite the recommendations of the IASSID in 1998, SeeAbility (2016) reported of the children they tested, 43% had no history of eye care, despite being 28 times more likely to have a serious sight problem than other children. NHS England (2019) acknowledging this unmet need, agreed to fund a national eye care pathway to provide regular sight tests for children in special schools within the next five years as part of the NHS Long Term Plan. Individuals with LD over the age of 19 years, however, are still at significant risk of dropping out of the eye care system which is exacerbated by a current lack of national monitoring of people with LD who have VI in the UK. Emerson and Roberston (2011) highlighted the large gap between the estimated number of adults with LD and VI/blindness known to services (42,000 adults) versus estimates of those not known to adult health or social care services (55,000 adults), and predicted the incidence was likely to increase by approximately 0.5% each year for the next 20 years.

Government White Papers ‘Valuing People’ and ‘Valuing People Now’ (Department of Health, 2001, Department of Health, 2009) state that all people with a LD should access routine sight checks at their local optometrist. However, for those people with S/PLD, specialist services are required (Starling et al., 2006; van Splunder et al., 2004; Woodhouse, Griffiths & Gedling, 2000); as Allied Health Professionals (AHPs), orthoptists are ideally placed to provide functional vision assessments and to play a coordinator role between primary and secondary care.

People with LD have, to an extent, avoidable sight loss and are at risk of being marginalised. These differences in health status compared to individuals with no disabilities represent a health inequality (Marmot, 2010). The rationale to provide a service in South Devon required evidence of an unmet need at a local level. A cross reference between the local LD register and the register of blind and partially sighted people in Devon in 2003 revealed that of 1,866 people with LD known to services at that time, only 34 (1.82%) were registered as VI. (Skilton, 2003). Current estimates indicate a total adult LD population for South Devon of 5257, of which 403 are estimated to be sight impaired and 132 are estimated to be severely sight impaired/blind. These figures are calculated using the PANSI online tool utilising the VI and blindness prevalence rates for adults with LD of 5% and 2%, respectively, for 18–54 year olds and 11% and 3%, respectively, for 55yrs+ (PANSI, 2019). Research suggests these predicted figures represent a significant underestimation of the actual unmet need. Therefore, the aim of this service is to improve access to eye care for adults with learning disabilities in South Devon.

## Method

The initial pilot study was carried out in 2006 and led to the current service being introduced in 2007. Details of subsequent service set-up and an analysis of a five-year sample of people receiving an orthoptic home visit (OHV) between 1.1.2014–31.12.2018 are included. It was agreed by the local Research & Development and Information Governance departments that both the initial pilot study and this report constitute service evaluations and therefore do not require ethical approval.

In 2006, 81% of the pilot sample (n = 37) of LD individuals assessed by the orthoptist required further follow up for their eye care. 10% were certified as VI and 13% of people were identified as unable to access routine sight tests with their local optometrist. The study’s report outlined the outcomes, recommendations, and service user feedback following this pilot (Diplock, Smith & Skilton, 2007). These findings were disseminated both locally and nationally and resulted in an invitation from our local Primary Care Trusts – Torbay & Teignbridge – for the orthoptist to undertake an extended role in developing and coordinating a local eye care pathway for adults with LD. This included identifying key barriers to accessing eye care and implementing strategies to improve. The principal objectives were as follows.

### Objective 1: To Raise awareness locally and nationally

This is vital as people with LD are reliant on their relatives/support workers to arrange their health checks and many people still believe eye tests are not possible for people with S/PLD. One of the first tasks was the production of local easy read materials, for example the ‘Getting My Eyes Checked’ leaflet: *https://www.torbayandsouthdevon.nhs.uk/uploads/23994.pdf*. This provides a point of contact whilst informing carers that every individual, irrespective of their ability, can have an eye test.

### Objective 2: Creation of a multi-disciplinary team (MDT) network

Creation of a MDT network is vital, including primary care liaison nursing teams, optometrists, sensory teams, Diabetic Eye Screening (DES) programmes, consultant ophthalmologists, nurses from the hospital eye clinic, and local LD commissioners. This allows the orthoptist to engage local stakeholders–especially in primary care–to ensure regular, accurate referrals, and raise awareness amongst AHPs and community LD teams of the importance of considering referral for vision assessment prior to treatment planning.

As there is no central community LD centre in Torbay, the MDT network liaise via secure email and all members of the team input to the service as and when required as part of their work remit. The orthoptist has an extended role (Band 7-Advanced Orthoptist) 0.2 whole time equivalent to deliver this service. This role includes coordination of the service, OHV’s, and chairing a service review meeting twice a year to set time-limited action plans. This maintains eye care as a top priority for the MDT and ensures momentum of the service. Due to the complexities of the adult pathway, a targeted approach is required. Only those people that require additional support are referred and wherever possible people with mild/moderate LD are encouraged to access routine sight tests with their local optometrist.

Desensitisation pathways have dramatically improved access to eye care for local people with S/PLD through the use of tailored plans. These include a course of pre-appointment visits to familiarise a person with the eye clinic setting and to identify any reasonable adjustments required. Once the initial desensitisation programme has been carried out, future sessions tend to be much shorter. Close liaison with the local DES provider has ensured even those people with the most severe LD are able to access their annual diabetic eye screening.

### Objective 3: The Orthoptic Home Visit (OHV)

The pilot study identified a small group of people (13%) who required additional support. It was therefore agreed the service would provide an OHV for any adult with LD unable to access eye tests locally. The vast majority of people referred have S/PLD often rendering formal vision assessment challenging or impossible. Tests, where possible, include assessment of visual acuity (VA) and/or visual function using the test most appropriate for the level of the person’s LD; contrast sensitivity with Hiding Heidi cards; cover test; ocular motility in the 9 positions of gaze; confrontation fields (usually with both eyes open); gross eye health for signs of red eyes, eye infections, cloudy cornea, etc.; and red reflex. Binocular single vision potential and evidence of colour vision is assessed where cooperation/comprehension permits. Potential visual processing difficulties/CVI is assessed with the aid of a detailed question inventory adapted (utilising wording appropriate for adults) from the Visual Skills Inventory for Children (Dutton and Bax, 2010).

The nature of this service means that virtually everyone referred here lacks the capacity to consent. Eye care professionals have a duty to ensure all patients consent to assessment and treatment. Unlike children, adults (16 years or over) are deemed to have capacity to consent unless it can be proved otherwise, in which case a procedure may be undertaken in that person’s best interest (Mental Capactiy Act, 2005). This is clarified at the point of referral.

An integral part of the OHV is the outcome report which provides a detailed history, including a resume of any birth/family history, previous eye health and general health, in particular the presence of syndromes, diabetes or epilepsy and any medications. A baseline assessment of current functional vision levels, with support strategies where appropriate, and a clear outline of any actions required is also provided. Actions often include referral to the hospital eye clinic or discharge to a specialist optometrist for routine domiciliary sight tests. The SeeAbility easy read form ‘Telling the Optometrist About Me’ is used to tailor reasonable adjustments to meet individual needs (SeeAbility, 2020).

Several projects have now been developed in England, however these are limited and mainly centred in cities, for example, some London Boroughs, Greater Manchester and Bradford (SeeAbility, 2016). These services confirm findings that any national eye care pathway for adults with LD will require local adaptations to ensure access is truly equitable for those people with the most complex needs. Despite being locality specific, this service model has sufficient flexibility to allow integration into any future national eye care pathway.

## Results

The data for this service evaluation relates to all individuals assessed for OHVs during the five-year period 1.1.2014–31.12.2018 (N = 90 different people). Participants were aged between 17–79 years (mean ± SD, 42.5 ± 16.4), with a higher incidence of males: 54 (60%) compared with females: 35 (40%). Seventy-one individuals required a single visit. Those individuals receiving ongoing ophthalmology intervention or diabetic screening (N = 19) had multiple OHVs within this period resulting in a total of 118 OHVs.

***Table 1*** highlights the source of referrals. The majority were from primary care which is key to the success of the service.

**Table 1:** Source of referrals (N = 118) for an OHV.


	NUMBER OF REFERRALS FOR OHV	%

Community LD Primary Care Liaison Nurses	30	25.4

Community LD AHP’s	25	21.1

Orthoptists	16	13.5

Ophthalmologists	13	11

DES programme (annual reviews)	13	11

Home Managers	11	9.3

GPs	4	3.3

Relatives/key workers	2	1.8

Community optometrists	2	1.8

Other	2	1.8


The dramatic change in social care provision from institutions to supported living in the community, whilst infinitely preferable, has the potential for people to become isolated and miss out on health checks. Flexibility in OHV settings is therefore required (***Table 2***).

**Table 2:** Setting for OHV’s (N = 118 OHVs).


	NUMBER OF OHVS	%

Group supported living	54	45.7

Family home	29	24.5

Community resource centre	20	17

Local community hospital	8	6.7

Individual supported living	7	5.9


For older people, diagnoses are often vague and past medical history very limited. As outlined in ***Table 3***, the cause of the LD was unknown for thirty individuals.

**Table 3:** Diagnoses/Comorbidities of people (N = 90) seen for an OHV.


	NUMBER OF ADULTS	%

Epilepsy	44	48.8

Unknown cause	30	33.3

Autistic Spectrum Disorder	27	30

Down syndrome (DS)	22	24.4

Cerebral Palsy (CP)	17	18.8

Dementia	14	15.5

Other syndromes	12	13.3

Diabetes	11	12.2

Perinatal causes	7	7.7

Bilateral hearing loss with VI	4	4.4

Acquired brain injury	2	2.2


Owing to a significant proportion (74.5%) of individuals having a S/PLD, an accurate assessment of VA utilising Keeler 3m Crowded LogMAR test was only possible in 8.4% of participants (N = 10) and only 16.9% (N = 20) with the Linear Kay Picture test. Keeler Cards and Cardiff Acuity Cards both based on forced choice preferential looking techniques, utilising gratings, and vanishing optotypes respectively, were possible for 41.5% (N = 49). For the remaining 33%, white Stycar balls, graded by size, as well as brightly coloured toys were used to provide a baseline assessment of visual functioning in association with detailed questioning of the carers/relatives regarding visual behaviour.

The OHV outcomes as illustrated in ***Table 4*** confirm that, following service input, 41% (N = 49) of people were able to be discharged to specialist community optometrists for routine follow up, because support staff now had a better understanding of the person’s current vision levels and how to access sight tests.

**Table 4:** Outcomes from 118 OHV’s 2014–2018 inclusive.


	NUMBER OF OHVS	%

Discharge to specialist community optometrist	49	41.5

Referred to Hospital Eye Service (HES) Total	54	45.7

– Refer to HES for baseline assessment then discharge to optometrist	8	6.7

– Refer to HES Require treatment prior to discharge to optometrist	11	9.3

– Refer to HES Require regular on-going treatment/active monitoring	35	29.6

People requiring an Examination Under Anaesthetic	9	7.6

People provided with tailored advice	72	61

Referred from DES programme and successfully screened in HES.	11	12

Follow up orthoptic home visit	9	7.6

Deceased	13	14

Declined appointment	0	0


For those people referred to the HES (45.7%), interventions included glasses; cataract surgery; and treatments/active monitoring for corneal disease, diabetic retinopathy, and eye infections. For nine individuals an examination under anaesthetic was required, whilst for two people it was avoided by the use of desensitisation sessions. Additional tailored strategies were required for 61% (N = 72).

Ophthalmic findings for the study cohort are summarised in ***Table 5***.

**Table 5:** Ophthalmic findings for 90 people seen for an OHV 2014–2018 inclusive.


SAMPLE SIZE N = 90	AGE RANGE (YRS)	MANIFEST STRABISMUS	SIGNIFICANT REFRACTIVE ERROR	CATARACT ALL TYPES	NYSTAGMUS	KERATOCONUS	OPTIC ATROPHY	VISUAL IMPAIRMENT (VI)

S/PLD 74.5%	17–79	54% N = 49 ET 47% XT 51% Vertical 2%	41%	23%	14%	12%	3%	33%


### Refractive Error

Over the five-year period, 33.3% (N = 30) were prescribed glasses via a community optometrist, 32.2% (N = 29) were assessed and found not to require glasses, and for 17.7% (N = 16) the outcome of the optometrist test was unknown. Within the hospital setting, 7.7% (N = 7) were prescribed new glasses. For a further 8 people certified as VI, it was felt glasses would not improve vision. Prescriptions were issued for bilateral hypermetropia > +1.25DS, myopia > –1.25DS and astigmatism > 1.00DC. There was an extremely high incidence of astigmatism 75.6% (N = 28) associated with myopia (N = 18) and hypermetropia (N = 10). High refractive errors were common ranging from +5.75 to –24.50DS and –5.00DC. Only eleven individuals (12.2%) had been issued with a near addition.

### Oculomotor conditions

Overall, 54.4% (N = 49) of individuals had a manifest strabismus and ***Table 6*** demonstrates the preponderance of strabismus for DS and Cerebral Palsy (CP). The ‘other’ category included 13 people with a definitive diagnosis of either a specific syndrome or perinatal medical causes. Nystagmus was present in 14.4% (N = 13) of the cohort.

**Table 6:** Prevalence of manifest strabismus for different causes of LD.


		DEVIATION TYPE		

		ESOTROPIA	EXOTROPIA	VERTICAL DEVIATION

Down Syndrome	17/22 (77.2%)	14	3	0

Cerebral Palsy	7/17 (41.1%)	3	3 (1 Intermittent)	1

Other	26/51 (50.9%)	7	19 (3 Intermittent)	0


### Ophthalmic conditions

The service was introduced following evidence that only 1.82% of the local LD population were certified as VI. This figure has now risen to 26.6% with a further 6.6% declining certification (total of 33.2%). Cataracts were a primary cause of VI for 23.3% of the service users particularly for those people with DS, and a further 10% were pseudophakic. Keratoconus was present in 12.2% (N = 11) but for the 22 people seen with DS the incidence was much higher (27%). Optic atrophy was present in 3.3% (N = 3).

## Discussion

Fourteen years of service provision has highlighted several key factors which may be of value to other orthoptists seeking to set up a local service.

If a national eye care pathway is introduced, people with S/PLD/severe autism could still be marginalised unless the service is coordinated at a local level. Orthoptists are uniquely placed to provide this role, bridging the gap between primary and secondary care. In this way accessibility issues can be identified and remedied in a timely fashion. Eye care professionals may require additional training to carry out functional vision assessments whereas many orthoptists already have years of experience working with children with LD.

This service has been proactive in supporting the national campaign and highlighting the potential contribution of orthoptists as outlined in ‘Delivering an Equal Right to Sight’ (SeeAbility, 2016). The orthoptic coordinator for this study represented the British and Irish Orthoptic Society at the launch of the SeeAbility report in the House of Lords in 2016 and hosted an information gathering day for commissioners from NHS England to look at this service model in 2018. The service has also provided case studies for several key reports (Public Health England, 2020; RSPH, 2020; SeeAbility, 2016).

### Measuring outcomes

Having realistic aims allowed establishment of a service that was both practical and sustainable. As previously outlined, the aim of the service model is not to screen all adults with LD but to provide a safety net for those people with the most complex needs.

Measuring visual outcomes is particularly challenging for this group. Whilst the term ‘people with S/PLD’ is used throughout clinical and research settings, direct comparison of outcomes must be viewed with caution due to the heterogeneity of published studies, for example: Different ages, severity of the LD, different diagnoses, and existence/severity of comorbidities. Despite this, evaluation of OHV outcomes from this service show many similarities to previously published reports.

Accurate clinical assessment may not be possible, and many research results are skewed due to a significant proportion of people with S/PLD being omitted from study findings because they could not be assessed. Woodhouse, Griffiths & Gedling (2000) still found a significant difference in the level of VI between the mild-moderate and S/PLD groups even though the analysis excluded 18% of people with S/PLD who were unable to cooperate with testing.

Pilling, Outhwaite & Bruce (2016) developed a functional vision assessment tool, ‘The Bradford Visual Function Box.’ This was in response to their experience that children with severe LD lose attention/fail to respond to the monochrome stimuli of forced choice preferential looking tests. This service noted similar responses in adults with S/PLD, especially to grating acuity tests. Warburg (2001b) commented that resolution difficulties may present serious limitations in the use of gratings as a measure of VA for people with S/PLD. People with S/PLD may not respond to novel objects but often display excellent visual attention when presented with familiar silent objects. Roman-Lantzy (2007) lists difficulty with visual novelty as one of ten key behavioural characteristics for children with CVI. Flexibility therefore is the key, but documenting details of object size and colour is essential to achieve a repeatable measure of visual functioning. Guidance on assessment of functional vision and interpretation of results is available where formal assessment of vision is not possible (Li et al., 2015; Pilling, Outhwaite & Bruce, 2016; SeeAbility, 2020).

The results of this study demonstrate that delivery of eye care needs for people with S/PLD is possible, however, measuring the impact of these interventions on an individual’s quality of life (QoL) is challenging. In the absence of formal QoL questionnaires (which are not practical within the constraints of a home visit), the values of this service can be heard in the user feedback:

- It was very helpful to have an experienced person to give a fresh pair of eyes to B’s functional vision and reassuring that signs have not been missed.
- Kathy was so understanding and patient with my disability and provided knowledge about my eyes I have never had before. Awesome service – would recommend it.

### Refractive error

In this service, glasses were prescribed irrespective of the severity of the LD and were often trialled with strategies to build up wear. However, it became apparent that glasses were often not tolerated. This concurs with van Splunder et al. (2003) who noted that for people with S/PLD, VI was more likely to be associated with ocular abnormalities rather than refractive error (as found in those with mild/moderate LD). Other considerations included prescribing near additions for accommodative lag and presbyopia. Accommodative lag is known to be common in children with DS (> 75%), (Stewart, Margaret Woodhouse & Trojanowska, 2005) and CP (57.6%) (McClelland et al., 2006), even after the full correction of any refractive error. However, little is known about the impact of uncorrected accommodative lag in adults, and raises the possibility of including dynamic retinoscopy as part of the routine sight test for all young adults with LD. Routinely prescribing presbyopic prescriptions for older adults with S/PLD is also essential, but often not trialled.

### Oculomotor conditions

Prevalence patterns of strabismus for different causes of LD (***Table 6***) appear similar to research findings. For adults with DS, esotropia was found to be more prevalent than exotropia (not seen with other causes of LD) and was unusual in that it often co-existed with a varying degree of myopia/myopic astigmatism, also noted by Krinsky-McHale et al. (2012). This appears to be specific to the adult DS population, and unlike other causes of LD where deterioration in vision results in a tendency for the eyes to diverge, with DS the esotropia appears to persist.

For people with CP, in line with previous studies (Katoch, Devi & Kulkarni, 2007; Kozeis et al., 2007), the prevalence of manifest strabismus irrespective of the type of CP, showed a more even distribution of esotropia (17–26.2%) versus exotropia (22–27.7%). Fazzi et al. (2012) noted that people with tetraplegia showed a severe neuro-ophthalmological profile including ocular abnormalities, oculomotor dysfunction, reduced VA, and CVI. This has direct implications where eye-gaze based assistive augmentative technologies are being considered to aid communication, as the presence of poor VA, alternating strabismus, nystagmus, and CVI are listed as key barriers to utilising this type of system (Clarke, 2016). This service has provided baseline functional vision assessments to help inform this process.

### Ophthalmic conditions and CVI

The majority of the service users have S/PLD, and certification rates locally have now increased from 1.82% to 26.6%, however, the research evidence outlined above suggests that under-registration is still taking place. Possible explanations are that certification for VI offers little in the way of financial benefits and that access to expert advice via the sensory teams is available without it, hence there is often ambivalence towards the certification process for this group. However, it can provide benefits, by evidencing the extent and severity of VI to inform service provision and informing support workers that the individual in their care has a significant sight problem.

One of the main causes of severe sight impairment for the service users was bilateral optic atrophy. McCulloch et al. (1996) noted that optic atrophy was most prevalent in those with S/PLD, is frequently associated with retro-chiasmal visual pathway abnormalities, and therefore often occurs in association with CVI. Studies suggest that CVI may improve in some children with age (Good, 2001; Hoyt, 2007), however for many people, particularly for those with S/PLD, CVI persists into adulthood. Several studies have identified CVI as the most common untreatable condition for people with LD diagnosed with VI (van Splunder et al., 2004; Warburg, 2001b). In their review paper Watt, Robertson & Jacobs (2015) highlight evidence of changes in the visual cortex in people with DS in support of this. Despite that, there is a lack of discussion regarding how CVI impacts on visual functioning and QoL. For this study’s service, a definitive diagnosis was frequently unavailable, therefore the term ‘visual processing difficulties’ was used to ensure everyone can access advice strategies including people experiencing symptoms related to possible visual misperceptions associated with Alzheimer’s Disease (Alzheimer’s Society, 2020). Symptoms relating to visual processing difficulties were common and strategies were utilised in 61% of people. These strategies developed by McDaid, Cockburn & Dutton (2008), have the additional benefit of raising awareness amongst support staff of how visual processing difficulties can impact on a person’s day to day life and may provide an insight into previously unexplained challenging behaviours.

Cataracts formed the most common ophthalmic finding for our service users. This study’s service ensures that people with significant age-related cataracts are actively monitored and that treatment is initiated where appropriate, at the point deterioration in functional vision starts to impact on QoL. This service supports the recommendations for cataract surgery for people with LD outlined by Pilling and Rostron (2014). This emphasises the importance of the multidisciplinary approach and reasonable adjustments for surgery including pre- and post-operatively. On two occasions, the orthoptic coordinator organised and chaired ‘best interest’ meetings where capacity to consent was an issue.

The onset of keratoconus during adolescence poses a significant risk factor for some of the young service users with DS, due to the potential for their routine sight tests to stop when they move from child to adult services. Attempts to address this are made by ensuring that young people with DS are referred directly to this service and that the orthoptist makes an annual visit to the further education departments of local special schools to talk about the importance of regular sight checks.

It is well recognised that prevalence rates for Types I and II diabetes are higher for people with LD compared with the general population. The recent NHS RightCare (2017) pathway for diabetes confirms that people with LD are at higher risk of Type II diabetes and obesity (a risk factor for Type II diabetes). However, for people with DS, the higher prevalence rate is associated with Type I (insulin dependent) diabetes. By maintaining close links with the local DES provider, this service has seen uptake rates for DES for people with LD increase from 45% prior to our service to 93% in 2018.

During the studied time period, none of the patients had a diagnosis of glaucoma, despite its high prevalence in the general adult population (Li et al., 2015). However large population studies of adults with LD show low prevalence rates for glaucoma 1.1% (Warburg, 2001b) and 2% (Krinsky-McHale et al., 2012).

Neurodevelopmental comorbidities can have a considerable impact on a person’s ocular health, visual processing, and the ability to cooperate with testing. Severe autism, epilepsy and the onset of dementia were significant factors for people requiring an orthoptic home visit.

In this study 30% (N = 27) of patients had a diagnosis of autism. In a systematic literature review, Butchart et al. (2017) reported an incidence of 22.9–32.7% for refractive error and 8.3% for strabismus from a series of studies. They commented that many of the studies showed evolving evidence of increased incidence of ophthalmic disorders in Autistic Spectrum Disorder particularly where it is associated with LD. This study’s results support this finding with an incidence of manifest strabismus (22%).

Just under half of the study cohort had epilepsy/significant seizure activity (48.8%). Van den Broek et al. (2006), in examining VI in people with S/PLD, found those with epilepsy (71%) showed significant agreement between severity of VA and problems with visual attention. This study’s results are in agreement with this report as the majority of patients with epilepsy had nystagmus (69%) and strabismus (59%). This complexity of health issues can increase with age such that van Buggenhout et al. (1999) found 42% of people with DS over the age of 50 years had dementia, and, of this group, 50% also had epilepsy.

The dramatic increase in life expectancy for adults with DS is highly significant in that biological age often exceeds chronological age by approximately 20 years. A systematic review by Torr et al. (2010) highlighted age-related, early onset disorders resulting in functional decline in adults with DS. Not surprisingly, sensory impairments were particularly prevalent.

The review paper by McQuillan et al. (2003) confirmed adults with DS are at increased risk of Alzheimer’s Disease, the incidence increasing significantly with age. The average age of onset is estimated at 52 years with an average duration of less than 5 years. As a result, older people with DS are referred to this service to clarify whether a recent deterioration in function is due to dementia or a visual cause. The OHV report provides valuable information for the wider dementia screening process.

## Conclusion

People with learning disabilities have poorer health which, to an extent, is both avoidable and unjust (Ouellette-Kuntz, 2005), highlighting the health inequalities people with LD face. The development and delivery of this local service has identified a significant unmet need in eye care provision for people with S/PLD and provides a service model to address this.

This study’s cohort were referred to this service because they had not been accessing eye care. Many instances of positive clinical outcomes and improvements in QoL have been identified, highlighting the significant reduction in health inequalities that can be achieved at minimal cost. The current commitment by NHS England to fund an eye care pathway for children with LD and engage with stakeholders regarding a pathway for adults with LD is extremely positive.

There is already a wealth of research evidencing the unmet need. By providing the crucial link between primary and secondary care, orthoptists are ideally placed to liaise with members of the eye care team and enablers in adult social care to deliver joined up eye care that meets the needs of the individual. Orthoptists also possess the ideal skill set for carrying out the specialist functional vision assessments required by adults with S/PLD.

It is hoped that this service model will inspire other orthoptists to work proactively to identify areas of health inequality and raise awareness with stakeholders, in order to establish local initiatives. Potentially these could then be integrated into any future national framework proposal to ensure access to eye care is truly equitable for those people with the most complex needs.
